# Web-Based Narrative Game Intervention for Initiating Advance Care Planning Among Adults: Exploratory Cross-Sectional Feasibility and Acceptability Study

**DOI:** 10.2196/94395

**Published:** 2026-08-24

**Authors:** Takashi Iwashiro

**Affiliations:** 1 Kobe Institute of Computing, Graduate School of Information Technology, 2-2-7 Kano-cho, Chuo-ku, Kobe, Hyogo, 650-0001, Japan, 81 80-1483-2370

**Keywords:** advance care planning, interactive narrative, narrative medicine, feasibility study, psychological readiness, web-based intervention, digital health education, visual novel

## Abstract

**Background:**

Advance care planning (ACP) is an ongoing dialogical process. Despite growing policy-level recognition, ACP engagement remains limited in Japan, and many individuals remain unfamiliar with the concept. Beyond knowledge deficits, insufficient psychological readiness may also impede engagement. Evidence on fully online narrative- and game-based approaches to supporting such readiness remains limited.

**Objective:**

This study aimed to explore the feasibility and acceptability of a fully web-based ACP educational intervention integrating an explanatory video and an interactive game and to evaluate postintervention cognitive, attitudinal, and psychological responses.

**Methods:**

This exploratory cross-sectional online study was conducted in Japan between July and August 2025. The final analytic sample comprised 158 adults, including 46 with health care or long-term care experience and 112 without. Participants watched an animated explainer video, played an interactive narrative game, and completed a postintervention questionnaire online. Feasibility and acceptability were assessed based on participation and response distributions. Responses to 12 questionnaire items (Q7-Q18) were rated on a 5-point Likert scale. Between-group comparisons were conducted using 2-tailed Welch *t* tests (α=.05). Open-ended responses were organized into broad descriptive themes.

**Results:**

Participants' mean age was 44.4 (SD 11.5) years, and 36.1% (57/158) were female. Postintervention responses showed generally favorable engagement. Mean scores exceeded the scale midpoint (3.0) for most items, including improved understanding of ACP (Q8: mean 4.39, SD 0.75), perceived importance of ACP (Q13: mean 4.42, SD 0.68), and personal relevance (Q15: mean 4.42, SD 0.83). Responses showed greater variability for imagining a concrete ACP discussion (Q17: mean 3.72, SD 1.02) and perceived psychological barriers (Q18: mean 3.49, SD 1.17). Significant between-group differences were observed for Q7 (mean difference −2.49, 95% CI −3.01 to −1.97; *P*<.001), Q10 (mean difference −0.40, 95% CI −0.71 to −0.09; *P*=.01), Q16 (mean difference 0.30, 95% CI 0.00-0.60; *P*=.048), and Q18 (mean difference −0.94, 95% CI −1.33 to −0.55; *P*<.001).

**Conclusions:**

A fully web-based, asynchronous ACP intervention combining explanatory video and an interactive narrative game may support early educational strategies that foster reflection on ACP. By engaging participants as active decision-makers in a simulated environment, the intervention provides preliminary evidence for psychological readiness as an educational outcome distinct from immediate behavioral change. Controlled longitudinal studies are needed to evaluate sustained behavioral outcomes.

## Introduction

Advance care planning (ACP) is internationally recognized as an ongoing process through which individuals reflect on and communicate their values and preferences regarding future medical care [1,2]. Rather than being limited to advance directives or end-of-life documentation, ACP is aimed at supporting continuing dialogue among patients, families, and health care professionals throughout the life course [2]. Despite this conceptual shift, its implementation remains limited. In Japan, public awareness of ACP remains low—with a national survey reporting that 72.1% of citizens were unaware of ACP as of 2022 [3]—and ACP discussions are generally postponed until serious illness or hospitalization [4,5]. Previous studies have suggested that knowledge alone is insufficient to initiate ACP conversations and that multiple barriers—including psychological discomfort, prognostic uncertainty, and lack of preparedness—persist beyond simple information deficits [6,7].

A critical barrier in this regard is psychological readiness. Even when individuals understand the purpose of ACP, many find it challenging to perceive future health deterioration as personally relevant or to initiate conversations about such topics [8]. Research on risk perception has indicated that unrealistic optimism leads individuals to underestimate their own vulnerability, contributing to deferred engagement [9]. Caregiving experience and contextually grounded worry about future medical care have been identified as factors that promote ACP initiation, suggesting that psychological and experiential conditions—rather than knowledge alone—may be the key drivers of engagement [10,11]. Consequently, educational interventions designed solely to increase knowledge may have a limited impact on initiating ACP discussions [6,7].

Narrative-based learning and interactive educational games that simulate value-based decision-making have emerged as promising educational approaches because they encourage experiential engagement rather than passive information acquisition [12,13]. Narrative scenarios enable learners to project themselves into realistic situations and reflect on personal values, while interactive game elements actively involve participants in simulated decision-making [14,15]. Recent studies have demonstrated the feasibility, acceptability, and usability of game-based ACP education [16-18]. However, evidence regarding fully web-based, asynchronous ACP educational interventions remains limited, particularly with respect to participants’ cognitive, attitudinal, and psychological responses immediately following the intervention. In addition, although psychological readiness is considered an important prerequisite for engaging in ACP, it remains unclear whether digital educational interventions can effectively foster such readiness.

This exploratory study aimed to characterize participants’ cognitive, attitudinal, and psychological responses, following a fully web-based educational intervention integrating an animated explanatory video with a narrative-based interactive visual novel game designed to encourage reflection on ACP. Particular attention was given to psychological readiness for initiating ACP. As a secondary objective, postintervention responses were compared descriptively between participants with and without involvement in health care or long-term care to generate hypotheses for future controlled studies and to inform the design of scalable, accessible digital ACP educational interventions.

## Methods

### Study Design

An exploratory, cross-sectional design was employed to descriptively examine the distribution of cognitive, attitudinal, and psychological responses observed after exposure to a narrative-based digital intervention for ACP. The purpose was to explore how a combined educational experience, consisting of an explanatory video and an interactive narrative game, might be associated with initial postintervention responses in the context of the structural challenge of difficulty in initiating ACP discussions.

The study was designed within the framework of feasibility and exploratory intervention research, with a specific focus on assessing acceptability and identifying patterns of postintervention responses. “Effects” were operationally defined not as causal changes based on pre-post comparisons but as the distribution of responses observed following exposure to the intervention.

### Participants

#### Inclusion and Exclusion Criteria

The study targeted adults who were able to independently access the web-based intervention and complete an online questionnaire in Japanese. No restrictions were placed on age, gender, or occupational background. Responses were excluded if the survey verification code did not match the preregistered code list, if the code was inconsistent with the participant’s platform identification number, or if the record was inconsistent with the corresponding WordPress post view log. Responses with evidence of incomplete game completion were also excluded in accordance with the predefined data validation procedure.

#### Sampling Procedures

**Figure 1.**
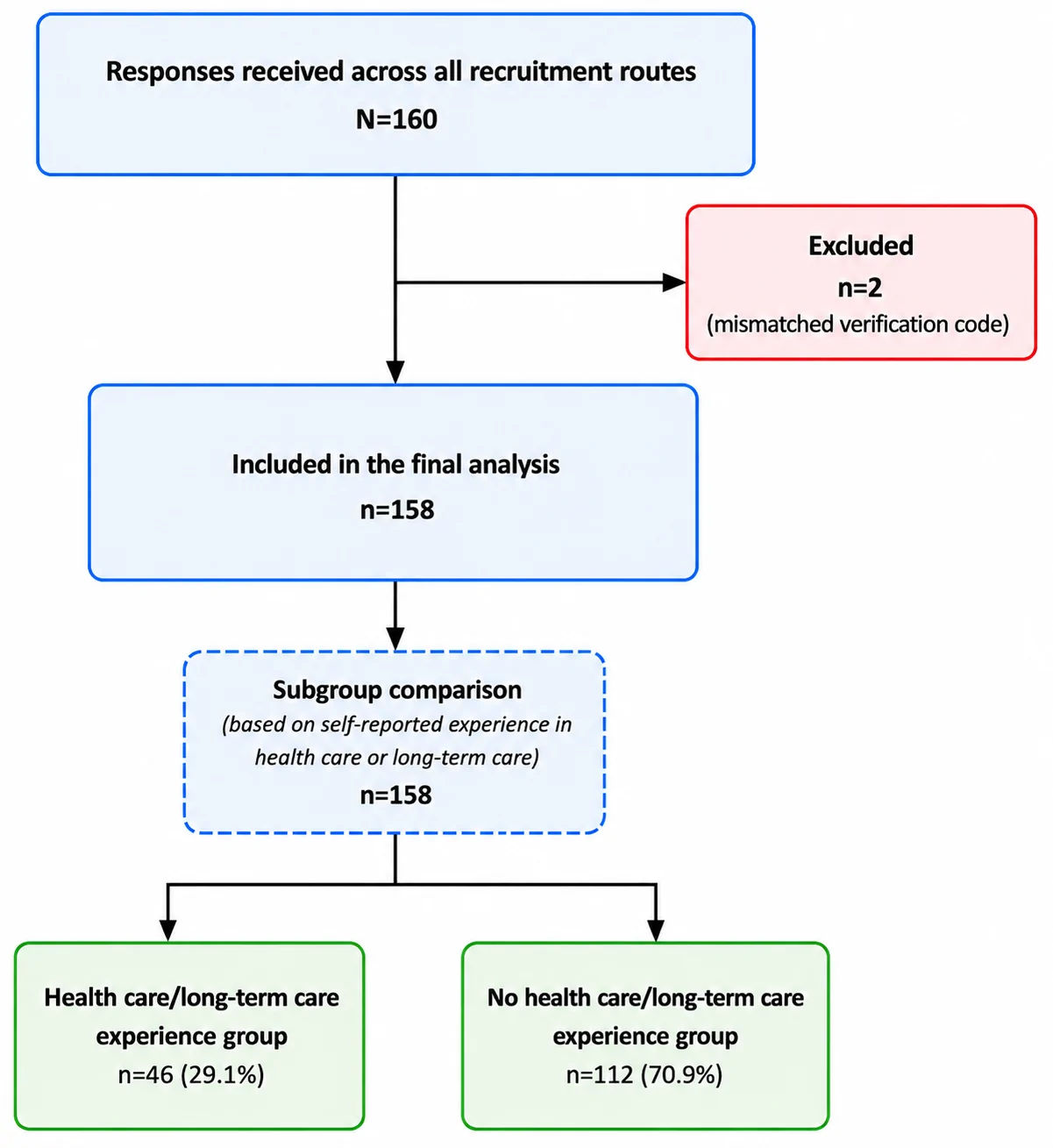
Participant flow diagram of the exploratory cross-sectional online study conducted in Japan between July and August 2025. Of 160 responses received across recruitment routes, 2 were excluded owing to mismatched verification codes, resulting in a final analytic sample of 158 participants. Participants were subsequently classified according to self-reported experience in health or long-term care for the exploratory subgroup comparison. Group classification was based on a self-reported item asking whether participants had any experience with health care or long-term caregiving contexts. Percentages may not total 100 owing to rounding.

Participants were recruited through 2 primary routes: individuals involved in health care or long-term care were recruited through the researcher’s professional network and with the cooperation of the Japan ACP Association, and individuals not involved in health care or long-term care were recruited via Lancers (Lancers Co), an online crowdsourcing platform. Crowdsourcing recruitment was conducted with a predetermined stopping rule, whereby data collection was closed once 100 valid responses were obtained for an adequate sample for exploratory descriptive analyses. A total of 160 responses were received across all recruitment routes; following the predefined validation procedure, 2 crowdsourcing responses were excluded owing to mismatched verification codes and were treated as incomplete intervention records, yielding a final analytic sample of 158 participants. The flow of participants through the study, including recruitment routes and exclusions, is illustrated in Figure 1.

#### Participant Characteristics

Participants were categorized into 2 groups for the secondary exploratory subgroup analysis: those with professional involvement in health care/long-term care and those without. A single self-reported item asking whether the participant had any personal experience with health care/long-term care—either through professional work or informal caregiving roles (“yes”=health care/long-term care experience group, “no”=no experience group)—was used for this classification. Specific occupational titles were not collected because the study was designed to examine experiential background broadly—defined as prior exposure to health-related decision-making contexts—rather than to compare participants by professional licensure or role. The final analytic sample comprised 158 participants: 46 in the health care/long-term care experience group and 112 in the no-experience group. Among the 112 participants in the no experience group, 98 were recruited through a crowdsourcing platform and 14 through referrals. Demographic characteristics of the sample are presented in Table 1.

**Table 1.** Demographic characteristics of participants (N=158)^a^.

Variable	Total (N=158)	Involved in health care/long-term care (n=46)	Not involved in health care/long-term care (n=112)
Age (y), mean (SD)	44.4 (11.5)	47.2 (12.8)	43.2 (10.8)
Gender: women, n (%)	57 (36.1)	25 (54.3)	32 (28.6)

a The table presents the characteristics of participants in an exploratory cross-sectional online study conducted in Japan between July and August 2025. Age categories were converted to numerical midpoints for analysis. One participant (included in the health care/long-term care group) did not report gender.

### Intervention

#### Overview of the Online Intervention

Participants accessed a dedicated web-based intervention page and completed the intervention sequentially in three stages: (1) viewing an animated explainer video with text-to-speech narration introducing fundamental concepts of ACP, (2) engaging in an interactive narrative novel game structured from the perspective of a patient’s family member, and (3) completing a postintervention questionnaire.

**Figure 2.**
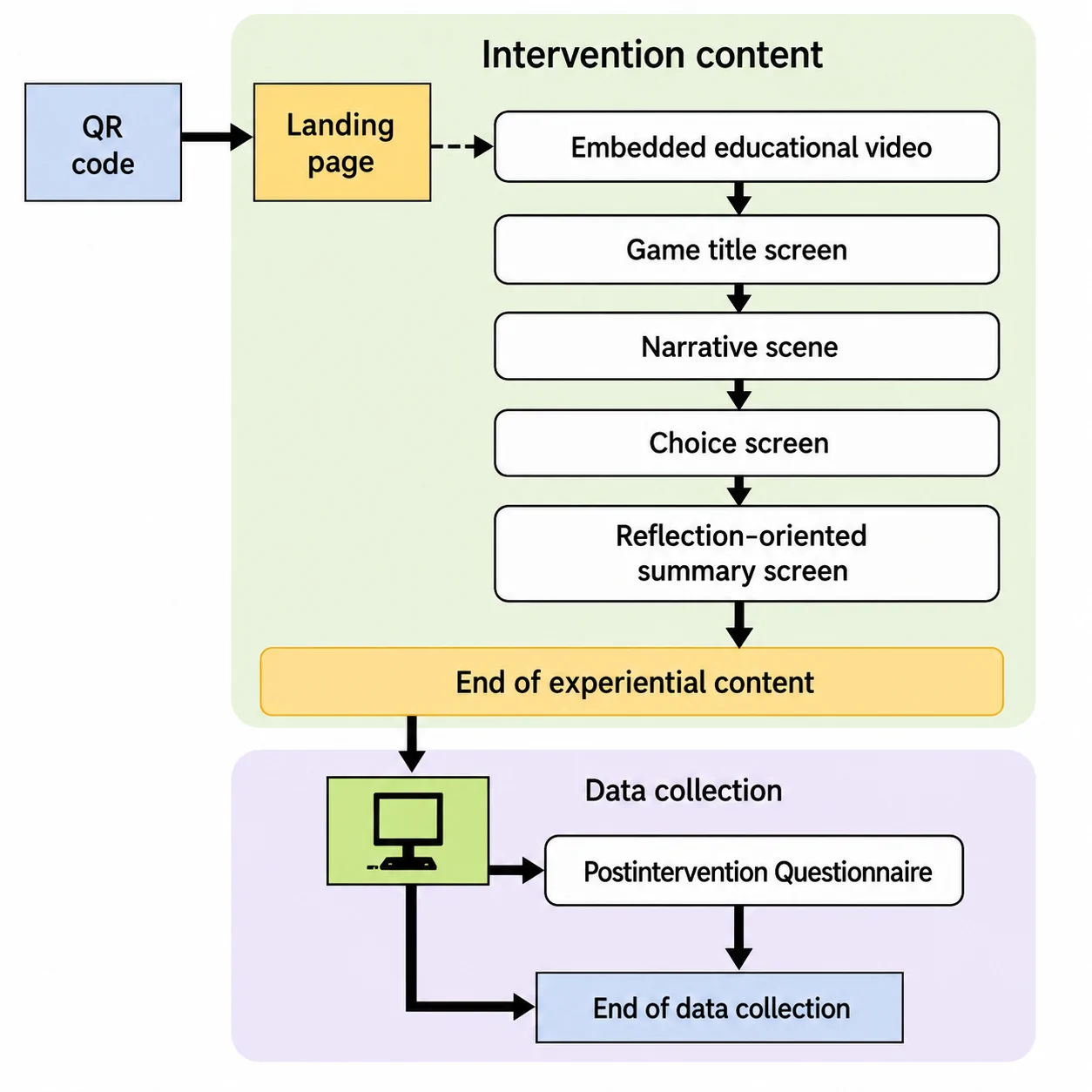
Structure and flow of the web-based advance care planning (ACP) educational intervention. Participants accessed the intervention through a landing page (via QR code), viewed an embedded educational video, and completed an interactive narrative game comprising narrative scenes, choice screens, and a reflection-oriented summary screen. After completing the experiential content, participants proceeded to the postintervention questionnaire for data collection in this exploratory cross-sectional online study conducted in Japan between July and August 2025.

All procedures were implemented remotely and asynchronously, allowing participants to access the intervention at a time and place of their choosing. Methodological reporting followed the CHERRIES (Checklist for Reporting Results of Internet E-Surveys) [19]. Ethical considerations regarding informed consent and privacy in web-based research were addressed in accordance with established guidelines [20]. Figure 2 depicts the intervention’s overall structure and flow.

#### Development of the Narrative Game

The composite ACP educational program was developed to progressively integrate cognitive understanding with emotionally engaged simulated experience. The instructional design was theoretically informed by experiential learning theory and prior research on narrative-based attitude formation and psychological engagement [21,22].

In the first stage, a prerecorded animated explainer video with text-to-speech narration, intended to support both conceptual understanding and the formation of psychological readiness, was employed. According to the cognitive theory of multimedia learning, human information processing relies on dual channels (auditory-verbal and visual-pictorial), each with limited capacity [23]. Combining narration and animation allows cognitive load to be distributed across channels (offloading), thereby facilitating comprehension [23].

The video was delivered asynchronously, enabling participants to pause and replay content at their own pace. Asynchronous video-based material supports self-regulated learning by enabling iterative viewing behaviors, such as pausing and rewatching, which are associated with improved conceptual understanding [24]. Considering that ACP generally involves emotionally sensitive themes linked to personal values and life perspectives [8], an asynchronous format was considered more appropriate than synchronous delivery, as it permits autonomous reflection without immediate social pressure.

In the second stage, participants engaged in a novel interactive narrative game structured around a scenario wherein a significant other suddenly faces a medical crisis. The narrative was designed to promote identification and perspective-taking and to foster emotional engagement through situated decision-making [12,13,22].

The educational prototype was independently developed by the author using Ren’Py (version 8.3.1), an open-source visual novel engine, and exported to a web format. The program was hosted on a secure server and made accessible through standard web browsers on both personal computers and mobile devices without requiring software installation. Choices made during gameplay were temporarily recorded solely for the purpose of generating the end-of-session “INITIAL DISCUSSION Summary” screen and were not retained as identifiable behavioral logs. No personally identifiable information was stored within the game environment.

Questionnaire responses were collected separately through Google Forms using an anonymous configuration, and all data were stored in password-protected institutional storage and analyzed in an anonymized form. The 2-stage structure combining informational presentation with simulated experiential engagement was designed to facilitate a shift from abstract knowledge acquisition toward personally contextualized reflection on ACP.

#### Structure and Interactive Design

**Figure 3.**
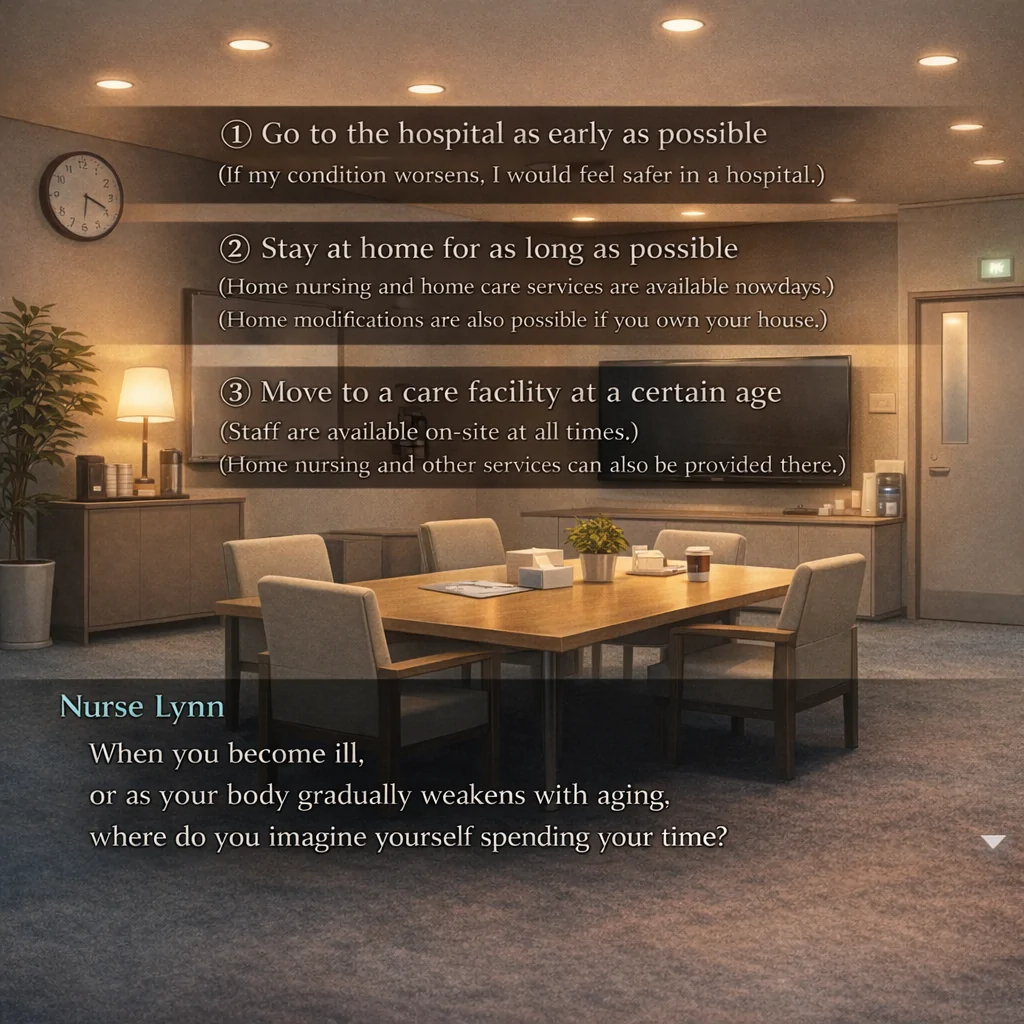
Example of the choice interface in the narrative-based interactive game employed in the web-based advance care planning (ACP) educational intervention. In this scene, participants are asked to reflect on future preferences for place of care and select one of several options presented within the narrative scenario. The interface was designed to prompt value-based reflection rather than to guide participants toward a predetermined answer in this exploratory cross-sectional online study conducted in Japan between July and August 2025.

The narrative game adopted an interactive structure in which multiple choices were presented throughout the progression of the story. Although the overall storyline and ending remained fixed, each decision point was designed to prompt participants to reflect on the characters’ values, interpersonal relationships, and decision-making processes. Through these structured prompts, participants were encouraged to engage in self-referential reflection rather than identify a “correct” answer. An example of the choice interface is shown in Figure 3.

The game did not provide explicit correct answers or evaluative feedback. This design reflects the study’s position that ACP should not be framed as an immediate decision-making task but rather as an ongoing process of reflecting on personal values. Previous research has suggested that narrative-based interventions incorporating active choice facilitate learner engagement by prompting reflection on decisions and their consequences [14]. Specifically, serious games that place participants in simulated dilemma situations have been reported to foster self-reflection and transformative learning [25].

Throughout the storyline, participants were presented with multiple ACP-related choices, such as preferences regarding life-sustaining treatment, their preferred place of care in the event of health deterioration, and messages they would like to leave for significant others. These choices were not intended to direct participants toward specific conclusions or behaviors but rather to allow them to articulate their thoughts at the time of engagement.

Immediately prior to the ending, selected responses were aggregated on a single screen labeled “INITIAL DISCUSSION Summary.” This screen displayed participants’ selected options, any free-text comments, and a timestamp indicating the time of completion. The summary functioned as both a reflective recap of the experience and a format suitable for screenshot capture.

The screenshot feature was designed in response to implementation challenges identified in ACP practice. Although conversations concerning ACP are widely recognized as valuable, the process of recording and making preferences accessible as personal records has been delineated as a core element of ACP [26]. Research on patients’ experiences with ACP has indicated that many face difficulty in translating the substance of conversations into written documentation [8], and international consensus definitions underscore the importance of regularly recording and revisiting expressed preferences over time [26].

**Figure 4.**
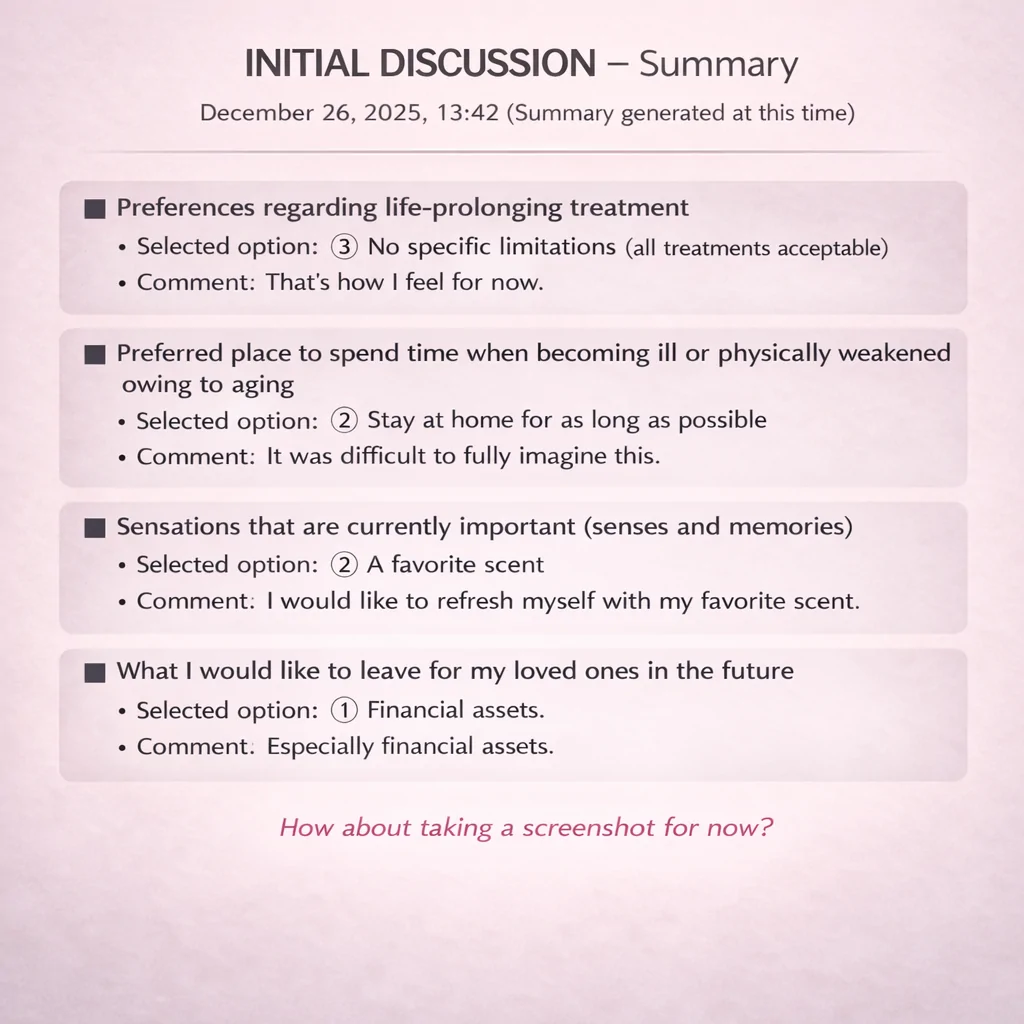
Example of the reflection-oriented summary screen displayed at the end of the narrative game in the web-based advance care planning (ACP) educational intervention. The screen aggregates the participant’s selected responses and optional comments generated during gameplay and presents them in a single view. This summary was designed to facilitate personal reflection on ACP preferences and could optionally be saved by participants as a screenshot for personal reference.

The summary was not intended to serve as a formal medical document or legal directive, but rather as a supplementary personal record enabling participants to retain their reflections at the time of engagement. Given that screenshot-taking is recognized as a normative behavior among young adults [27], this format was adopted as a pragmatic method to facilitate retention and optional sharing of the experience. An example of the summary screen is presented in Figure 4.

#### Procedure

After accessing the dedicated study web page, participants completed the intervention in the following sequence:

1. viewing a brief animated explainer video introducing the basic concepts of ACP (approximately 4 min);
2. completing the interactive narrative novel game (approximately 8 min); and
3. responding to a postintervention questionnaire administered via Google Forms (5‐10 min).

The system was configured such that participants could not proceed to the questionnaire unless the narrative game was completed. Partial completion of the game did not grant access to the survey. After completing the game, a unique survey code was automatically generated. Participants were required to enter this code into a designated questionnaire field to finalize their responses. For participants recruited via crowdsourcing, compensation eligibility was determined by verifying the survey code in accordance with the predefined data validation procedure described above. The intervention was conducted entirely online and asynchronously, allowing participants to access the content at a time and location of their choosing. The total duration of participation was 17 to 22 minutes.

#### Measures and Covariates

A 20-item self-administered online questionnaire was used for evaluation. Professional involvement in health care/long-term care served as the grouping variable for the secondary exploratory subgroup analysis. As the primary analyses were descriptive in nature, no covariates were included in the statistical models, and the grouping variable was employed solely for exploratory subgroup comparisons.

#### Primary Outcome Measures

To assess cognitive, attitudinal, and psychological responses formed after the intervention, 12 items (Q7-Q18) were designated as the primary outcome measures. Each item was rated on a 5-point Likert scale ranging from 1 (“strongly disagree”) to 5 (“strongly agree”). These items were designed to capture multiple dimensions related to ACP, including

- deepened understanding of ACP;
- attitudinal orientation toward ACP;
- future behavioral intentions regarding ACP-related discussions;
- perceived psychological barriers to engaging in ACP; and
- ability to imagine ACP as personally relevant in future situations.

#### Operationalization of Psychological Readiness

Psychological readiness was operationalized as a multidimensional pattern reflected in participants’ responses to items assessing (1) the ability to imagine ACP discussions in concrete situations (Q17), (2) perceived personal relevance (Q15), (3) intention to engage in ACP-related discussions (Q16), and (4) lower perceived psychological barriers (Q18). These indicators were interpreted descriptively and were not combined into a composite score, consistent with the exploratory design.

#### Open-Ended Item

Item Q20 invited participants to provide free-text comments regarding their impressions of the intervention and any perceived changes in their thinking. These qualitative responses were treated as exploratory material to supplement the quantitative findings.

#### Positioning of the Measures

No previously validated scales were used. Instead, consistent with early-phase item generation approaches in which items are developed prior to formal scale validation [28], the measures were constructed as operational indicators to examine the distribution of postintervention responses, reflecting the descriptive aims of feasibility studies [29].

#### Sample Size, Power, and Precision

As this study was exploratory and descriptive in nature, no a priori power analysis was conducted to determine sample size. The target sample size for participants recruited via the crowdsourcing platform was set at 100 valid responses to obtain an adequate sample for exploratory descriptive analyses. A predetermined stopping rule was applied exclusively to this recruitment route to obtain a reasonable number of responses for preliminary descriptive estimation in an exploratory context. Accordingly, the analyses were intended to provide descriptive and exploratory estimates rather than hypothesis-testing inferences. No formal power calculations or precision estimates were applied.

### Data Analysis

For quantitative analysis, means and SDs were calculated for each of the 12 primary outcome items (Q7-Q18) to describe the distribution of postintervention responses. As a secondary exploratory component of the analysis, postintervention responses were also descriptively compared between participants with and without involvement in health care/long-term care to examine preliminary patterns associated with professional background.

Owing to unequal group sizes, Welch *t* tests were conducted to compare responses between participants involved in health care/long-term care and those who were not involved. This comparison was performed as an exploratory subgroup analysis based on the premise that prior experiential exposure to health care or caregiving contexts may influence the formation of psychological readiness for ACP engagement [10,11].

Statistical significance was set at a 2-tailed α level of .05. As this study was exploratory and hypothesis-generating in nature, no adjustment for multiple comparisons was applied; findings from between-group analyses should therefore be interpreted as descriptive patterns rather than confirmatory statistical evidence.

Open-ended responses (Q20) were treated as exploratory supplementary data to contextualize and illustrate the quantitative findings. Responses were reviewed in their entirety and organized into broad descriptive themes based on recurring content patterns; representative excerpts were then selected from each theme to illustrate the range of reactions observed following the intervention. Although this was not designed as a formal mixed methods study, the integration of quantitative results with qualitative excerpts is theoretically supported as a complementary analytic approach [30].

### Ethical Considerations

This study was determined to constitute educational practice research and therefore was considered outside the scope of the Ethical Guidelines for Medical and Biological Research Involving Human Subjects issued by the Ministry of Education, Culture, Sports, Science and Technology, the Ministry of Health, Labour and Welfare, and the Ministry of Economy, Trade and Industry of Japan [31].

The study did not constitute medical or biological research involving human subjects. Accordingly, formal review and approval by an institutional review board were not required.

In accordance with Article 6 of the Research Ethics Regulations of the Kobe Institute of Computing, the study was reviewed and approved by the Dean of the Graduate School (acting as the Compliance Officer) and the supervising faculty member prior to data collection. No formal institutional review board approval number was issued under this institutional review process.

Participation was entirely voluntary. Before accessing the intervention, individuals received clear information regarding the study’s purpose, procedures, and their right to withdraw at any time without disadvantage, which was presented on a dedicated instructional webpage. Completion of the postintervention questionnaire was regarded as the provision of informed consent, consistent with established ethical practice in anonymous web-based research [19,20].

Personally identifiable information, such as names, contact details, or institutional affiliations, was not collected at any point. Questionnaire responses were collected using an anonymous Google Form. All collected data were stored in password-protected storage managed solely by the investigator, analyzed in anonymized form, and used solely for research purposes. Data were not disclosed to third parties.

Participants recruited via the crowdsourcing platform (Lancers) received ¥300 (approximately US $2) each, which was determined in accordance with the platform’s standard remuneration policy and considered commensurate with the estimated task duration (17‐22 min). Individuals were recruited through the researcher’s professional network or via direct referral participated voluntarily without compensation.

A separate small-group session was conducted with 4 undergraduate and graduate nursing students for the purpose of content review and intervention feedback. These participants received a token honorarium; however, their responses were not included in the primary analysis.

The narrative game included a summary screen (“INITIAL DISCUSSION Summary”) displayed upon completion of gameplay. This screen aggregated participants’ in-game selections and was designed to be optionally captured as a personal screenshot. The summary screen was automatically cleared upon session termination, and no images or screenshots were collected by the research team. Choices recorded during gameplay were retained only temporarily to generate this summary and were not stored as identifiable behavioral data. No identifiable participant images, screenshots, or other visual materials were collected by the research team or included in the paper or supplementary materials. Any screenshots or illustrative images presented in this paper do not contain personally identifiable information and cannot be used to identify individual participants [20].

Furthermore, the characters depicted in the game interface (Figures 3 and 4), including “Nurse Lynn,” are fictional characters created solely for the educational intervention and do not represent the study investigator, research staff, or any study participants. The summary screen shown in Figure 4 displays only the player’s in-game selections and optional free-text comments. No participant names or other personally identifiable information are displayed or stored.

## Results

### Participants and Data Overview

As described in the *Methods* section, 158 valid responses were included in the final analysis (46 with health care/long-term care experience and 112 without). All 12 primary outcome items (Q7-Q18) were administered as mandatory fields, and no missing data were observed for these variables. Demographic characteristics are summarized in Table 1.

### Overall Descriptive Results of Postintervention Responses

#### Descriptive Statistics for Questionnaire Items

Descriptive statistics for the primary outcome items (Q7-Q18) are presented in Table 2. Across all participants, the mean scores exceeded the neutral midpoint of the 5-point Likert scale (3) for several items, including understanding of ACP (Q8), perceived importance of ACP (Q13), and clarity of the educational content (Q10).

Items related to perceived psychological barriers (Q17 and Q18) had relatively larger SDs than those of the other questionnaire items (Table 2).

**Table 2.** Descriptive statistics of postintervention questionnaire responses (Q7-Q18; N=158)^a^.

Item	Description	Mean (SD)
Q7	I learned the term “ACP^b^” for the first time.	4.18 (1.50)
Q8	My understanding of ACP improved.	4.39 (0.75)
Q9	I was able to imagine specific situations for ACP discussions (eg, with whom and when to talk).	4.01 (0.83)
Q10	The educational materials (video+game) were easy to understand.	4.26 (0.85)
Q11	The story and presentation of the materials were memorable.	4.08 (0.87)
Q12	I was able to reflect on ACP in relation to myself.	3.93 (0.96)
Q13	I felt that ACP is an important issue related to quality of life.	4.42 (0.68)
Q14	I would like to learn more about ACP.	4.04 (0.87)
Q15	I felt that ACP is personally relevant to me.	4.42 (0.83)
Q16	I would like to discuss ACP with my family or loved ones.	4.05 (0.88)
Q17	I was able to imagine actually having an ACP discussion.	3.72 (1.02)
Q18	I felt psychological barriers to discussing ACP.	3.49 (1.17)

a These responses were obtained following the web-based narrative game intervention for advance care planning in an exploratory cross-sectional online study conducted in Japan between July and August 2025. All items were rated on a 5-point Likert scale (1=“strongly disagree” to 5=“strongly agree”). Descriptive statistics are presented for all participants (N=158).

b ACP: advance care planning.

#### Exploratory Group Comparisons by Professional Background

Welch *t* tests were conducted to compare responses between participants involved in health care/long-term care and those not involved in these fields (Table 3). In these exploratory comparisons, differences at the α level of .05 were observed for Q7 (*P*<.001), Q10 (*P*=.02), Q16 (*P*=.048), and Q18 (*P*<.001). No statistically significant differences were found for the remaining items.

**Table 3.** Exploratory group comparisons of postintervention questionnaire responses (Q7-Q18) between participants with and without health care/long-term care experience (N=158)^a^.

Item	Involved in health care/long-term care (n=46), mean (SD)	Not involved in health care/long-term care (n=112), mean (SD)	Mean difference (95% CI)	*P* value
Q7: I learned the term “ACP^b^” for the first time.	2.41 (1.72)	4.90 (0.40)	−2.49 (−3.01 to −1.97)	<.001^c^
Q8: My understanding of ACP improved.	4.15 (1.09)	4.48 (0.54)	−0.33 (−0.67 to 0.01)	.06
Q9: I was able to imagine specific situations for ACP discussions (eg, with whom and when to talk).	4.07 (0.80)	3.98 (0.84)	0.09 (−0.19 to 0.37)	.56
Q10: The educational materials (video + game) were easy to understand.	3.98 (0.95)	4.38 (0.77)	−0.40 (−0.71 to −0.09)	.02^d^
Q11: The story and presentation of the materials were memorable.	3.87 (0.98)	4.16 (0.81)	−0.29 (−0.62 to 0.04)	.08
Q12: I was able to reflect on ACP in relation to myself.	3.85 (1.01)	3.96 (0.94)	−0.11 (−0.46 to 0.24)	.50
Q13: I felt that ACP is an important issue related to quality of life.	4.43 (0.65)	4.42 (0.69)	0.01 (−0.22 to 0.24)	.90
Q14: I would like to learn more about ACP.	4.11 (0.97)	4.01 (0.82)	0.10 (−0.22 to 0.42)	.54
Q15: I felt that ACP is personally relevant to me.	4.46 (0.84)	4.41 (0.83)	0.05 (−0.24 to 0.34)	.76
Q16: I would like to discuss ACP with my family or loved ones.	4.26 (0.83)	3.96 (0.89)	0.30 (0.00 to 0.60)	.048^d^
Q17: I was able to imagine actually having an ACP discussion.	3.85 (1.07)	3.67 (1.00)	0.18 (−0.19 to 0.55)	.34
Q18: I felt psychological barriers to discussing ACP.	2.83 (1.16)	3.77 (1.06)	−0.94 (−1.33 to −0.55)	<.001^c^

a These responses were obtained following the web-based narrative game intervention for advance care planning in an exploratory cross-sectional online study conducted in Japan between July and August 2025. Group differences were examined using Welch *t* test. Values are presented as mean (SD). Mean differences represent the difference between the health care/long-term care group and the nonhealth care/long-term care group. All items were rated on a 5-point Likert scale (1=“strongly disagree” to 5=“strongly agree”). Significance markers are presented for descriptive purposes only, because these comparisons were conducted as a secondary exploratory analysis without a priori hypotheses and no correction for multiple comparisons was applied; therefore, the reported *P* values should not be interpreted as confirmatory statistical evidence. correction for multiple comparisons

b ACP: advance care planning.

c *P*<.001.

d *P*<.05.

#### Distribution of Responses Related to Psychological Barriers

**Figure 5.**
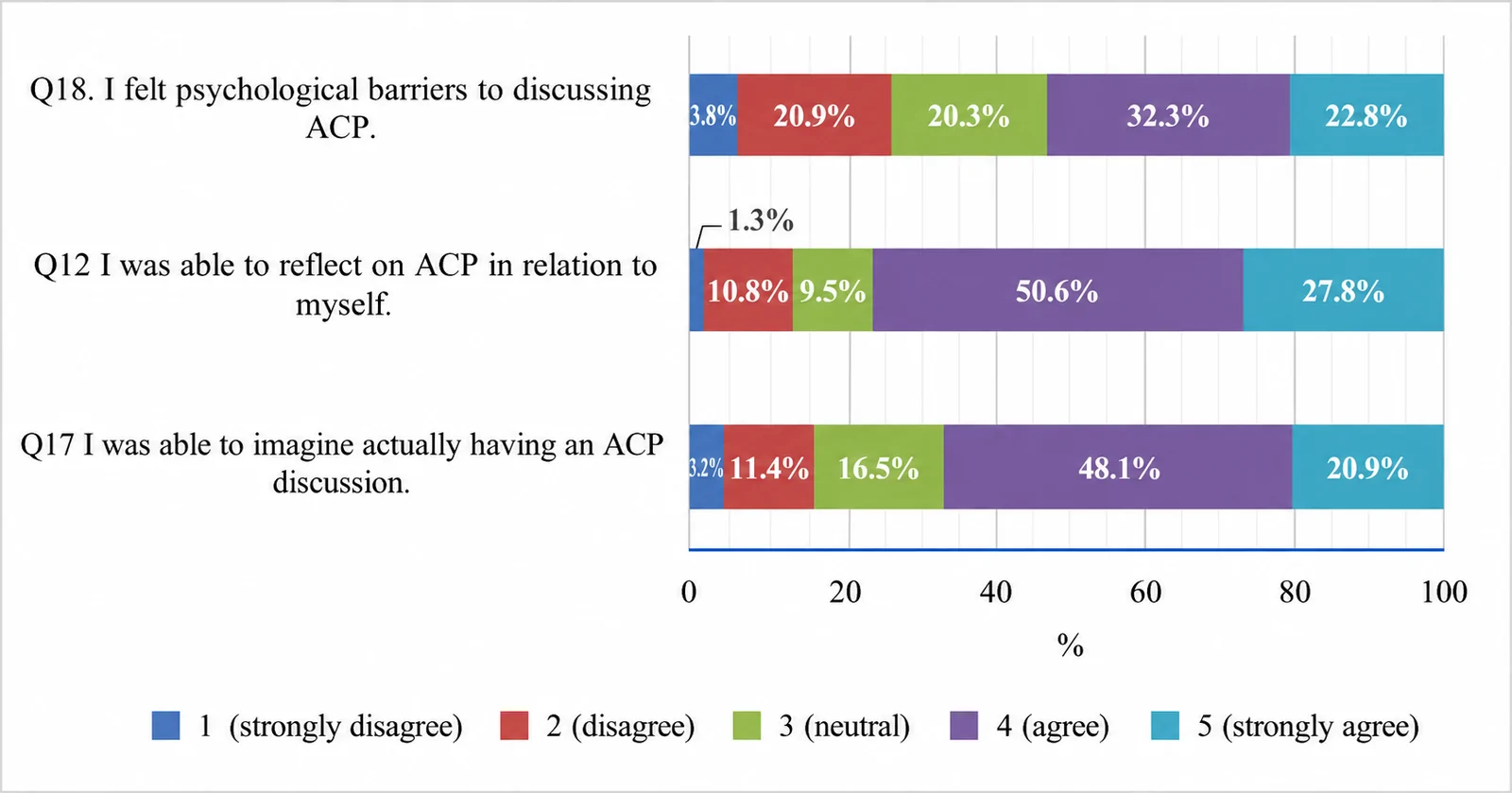
Distribution of responses to selected items related to psychological readiness following the web-based narrative game intervention for advance care planning (ACP) in an exploratory cross-sectional online study conducted in Japan between July and August 2025 (N=158). Stacked bar charts show the percentage distribution of responses for Q12 (self-referential reflection), Q17 (ability to imagine a concrete advance care planning discussion), and Q18 (perceived psychological barriers). Note. Response categories correspond to a 5-point Likert scale (1=“strongly disagree” to 5=“strongly agree”). Q18 is negatively worded; higher scores indicate greater perceived psychological barriers.

For items related to perceived psychological barriers (Q17 and Q18), response distributions were not concentrated at a single scale point but were distributed across neutral and positive categories (Figure 5).

#### Supplementary Qualitative Findings

To complement the quantitative results, representative responses from the open-ended item (Q20) are presented in Table 4. These excerpts do not constitute the results of a formal qualitative analysis but are provided as illustrative examples of reactions observed following the intervention.

**Table 4.** Representative excerpts from open-ended responses (Q20) following the web-based narrative game intervention (N=158)^a^.

Theme	Representative excerpt
Beginning to perceive ACP^b^ as personally relevant	“I understood that there may come a time when I cannot express my wishes, but seeing it presented in this way made me feel that I need to think about it as something that concerns me personally.”
Feeling that immediate decisions are not necessary	“I realized that it is not something that must be decided right away, which made me feel somewhat relieved.”
Increased awareness of preparing for the future	“I strongly felt that I need to start preparing from now. I am glad that I was able to learn about ACP in this way.”
Reduced psychological barriers to discussing ACP	“I began to feel that it would be okay to talk about it eventually, and it no longer felt as heavy as before.”
Impact of narrative elements and professional perspectives	“Hearing the nurse explain based on real experiences made it easier to imagine the situation in a realistic way.”

a These responses were obtained following the web-based narrative game intervention for advance care planning in an exploratory cross-sectional online study conducted in Japan between July and August 2025. Responses were reviewed and organized into broad descriptive themes based on recurring content patterns. Excerpts are presented as illustrative examples and do not represent the results of a formal qualitative analysis.

b ACP: advance care planning.

## Discussion

### Principal Findings

This exploratory cross-sectional study evaluated the feasibility, acceptability, and postintervention response patterns of a fully web-based, narrative-based ACP educational intervention integrating an explanatory video and an interactive visual novel game. The intervention was successfully delivered in a fully asynchronous online format and was broadly acceptable to participants, consistent with the objectives of feasibility-oriented research [29]. Overall, participants reported positive responses regarding their understanding of ACP, the perceived importance of ACP, and its personal relevance. Responses related to imagining future ACP discussions and to perceived psychological barriers exhibited greater variability, suggesting differences in psychological readiness to engage with ACP. Exploratory subgroup analyses revealed only limited differences between participants with and without prior involvement in health care or long-term care. Because this study did not include baseline measurements or a control group, these findings should be interpreted as descriptive postintervention response patterns rather than evidence of causal effects or behavioral change [29].

### Interpretation and Comparison With Previous Studies

ACP has increasingly been conceptualized as a continuing process of dialogue rather than a single decision-making event [1,26]. Nevertheless, previous studies have consistently reported that initiating ACP remains challenging because discussions are frequently delayed until medical crises occur and are influenced by emotional burden, uncertainty, and avoidance of future health deterioration [4,5,8,32]. The present findings align with this literature. Participants generally responded positively to the intervention, whereas responses related to psychological readiness demonstrated greater variability than those related to understanding of ACP or perceived importance of ACP. This pattern suggests that although participants readily understood the educational content, readiness to imagine engaging in future ACP discussions differed among individuals. Such variability aligns with previous descriptions of ACP as a highly individualized and emotionally sensitive process involving personal values and uncertainty regarding future health situations [1,8]. This observation also aligns with previous reviews suggesting that educational interventions improving knowledge and attitudes may be insufficient, on their own, to promote ACP engagement [6,7].

The intervention was intentionally designed to combine conceptual understanding through an explanatory video with emotionally engaging simulated experiences delivered through an interactive narrative [14]. This sequential structure reflects previous theoretical perspectives, suggesting that narrative experiences facilitate self-projection and personal meaning-making by transforming abstract health information into emotionally grounded reflection [12,13]. Rather than presenting ACP as a task requiring immediate decisions, the intervention encouraged participants to begin reflecting on their own values within a psychologically safe simulated environment, an approach consistent with narrative medicine and experiential learning frameworks [21,33,34].

Previous ACP educational interventions have largely relied on video decision aids or facilitator-led discussions [35,36]. Although such interventions have demonstrated improvements in ACP documentation and communication, participants have generally remained information recipients rather than active participants in simulated decision-making. In contrast, recent visual game-based ACP interventions have primarily been implemented in facilitated or synchronous formats and have focused on usability and immediate discussion outcomes [14,16-18].

The present intervention extends this growing body of research by evaluating a fully web-based, entirely asynchronous interactive educational program that integrates an explainer video with a branching narrative visual novel game and generates a personal “INITIAL DISCUSSION Summary” record designed to support ongoing ACP conversations without real-time facilitation. This design differs from previous game-based ACP interventions by positioning participants as decision-makers within emotionally engaging family-centered scenarios and by emphasizing psychological readiness as an educational outcome rather than immediate documentation or behavioral change [12-14,16-18,22].

Although these design features may partly explain the observed response patterns [12-14], the postintervention responses cannot be causally attributed to the intervention itself because no preintervention measurements or control group were included. Accordingly, it remains speculative whether the narrative design contributed to participants’ perceptions of personal relevance and psychological readiness or whether these responses reflected preexisting participant characteristics. Nevertheless, the observed response patterns align with the conceptualization of ACP as an ongoing reflective process rather than an intervention intended to produce immediate behavioral change.

Furthermore, the limited differences observed between participants with and without health or long-term care experience may suggest that narrative-based experiential learning operates beyond professional knowledge alone [37]. Previous qualitative studies have emphasized that ACP engagement is strongly influenced by personal relationships, caregiving experiences, and psychosocial contexts rather than professional expertise itself [38]. Accordingly, the present findings may indicate that experiential narrative interventions have the potential to function as introductory educational resources across diverse populations [16-18,21]. Although these interpretations remain exploratory because the study was not designed to test explanatory mechanisms directly, they provide preliminary support for future investigations into psychological readiness as a target of ACP education.

### Limitations

Several limitations should be considered when interpreting these findings. First, because this study was designed as an exploratory feasibility and acceptability study [29], no baseline measurements were obtained, rendering it impossible to determine whether the observed responses represented changes from participants’ preexisting attitudes. Second, the absence of a control group precludes causal interpretation of the observed response patterns [29]. Third, because the intervention combined an explanatory video with an interactive narrative game, each component’s contribution could not be evaluated separately. Fourth, the study was conducted exclusively in Japan, and cultural attitudes toward family decision-making and end-of-life communication may limit generalizability to other settings [39]. Finally, recruitment through online platforms may have introduced sampling bias owing to the nonrepresentative nature of internet samples and the self-selection of participants [19], and the grouping variable did not distinguish between different types or durations of health care or caregiving experience. Furthermore, the exploratory subgroup analyses should be interpreted cautiously because the observed between-group differences were descriptive rather than confirmatory. The variability in responses related to psychological readiness and the generally similar response patterns observed across professional backgrounds cannot be interpreted as evidence that prior health care experience does not influence ACP engagement. These findings should, instead, be regarded as preliminary observations that warrant confirmation in adequately powered controlled studies [29]. Consequently, the present findings should be interpreted as preliminary exploratory evidence requiring confirmation in controlled longitudinal studies.

### Broader Implications for Practice and Education

The findings suggest that ACP education may be better conceptualized not solely as a strategy for producing immediate behavioral change, but as an educational process that supports the gradual development of psychological readiness for future ACP engagement [1,40]. Drawing on frameworks of nonformal and experiential learning, narrative-based educational interventions may provide opportunities to begin reflecting on personal values in advance of future health care situations, rather than requiring participants to make immediate decisions [12,13,21,33,34].

The limited differences between participants with and without health care experience further suggest that such interventions may be suitable for diverse populations, including members of the general public and students as well as health care professionals. Accordingly, fully web-based narrative interventions may complement existing ACP educational programs by functioning as accessible, entry-level experiences that initiate a reflective process and encourage subsequent dialogue with family members and health care professionals. Future work could adapt culturally contextualized videos and games for other populations and examine long-term changes in individuals’ perspectives on ACP as they age [1,21,37].

### Conclusions

This exploratory study suggests that a fully web-based, narrative-based ACP educational intervention may serve as an accessible entry-level educational strategy for supporting reflection on ACP. Rather than promoting immediate behavioral change, the intervention may contribute to developing the psychological readiness necessary for future ACP discussions through experiential narrative engagement. These findings provide preliminary evidence supporting further investigation of narrative-based digital education within ACP implementation research. Future controlled longitudinal studies are warranted to examine sustained educational and behavioral outcomes.

Building on previous ACP educational interventions that have relied on video decision aids and facilitator-led discussions, this study evaluated a fully web-based, entirely asynchronous program integrating an explainer video, a branching narrative visual novel game and a personal “INITIAL DISCUSSION Summary” record. By positioning participants as active decision-makers within emotionally engaging family-centered scenarios, the intervention was designed to foster experiential reflection on values rather than presenting ACP solely as informational content.

These design features may help expand ACP education beyond clinical settings by providing a scalable, self-directed, entry-level resource that is applicable to both members of the general public and health care students. In addition, conceptualizing psychological readiness as an educational outcome distinct from immediate behavioral change may inform future intervention design and implementation research aimed at supporting earlier engagement with ACP across the life course.

## Abbreviations

ACP: advance care planning
CHERRIES: Checklist for Reporting Results of Internet E-Surveys
